# Absence of contamination of personal protective equipment (PPE) by severe acute respiratory syndrome coronavirus 2 (SARS-CoV-2)

**DOI:** 10.1017/ice.2020.91

**Published:** 2020-03-26

**Authors:** Sean Wei Xiang Ong, Yian Kim Tan, Stephanie Sutjipto, Po Ying Chia, Barnaby Edward Young, Marcus Gum, Sok Kiang Lau, Monica Chan, Shawn Vasoo, Shehara Mendis, Boon Kiat Toh, Janice Leong, Timothy Barkham, Brenda Sze Peng Ang, Boon Huan Tan, Yee-Sin Leo, Kalisvar Marimuthu, Michelle Su Yen Wong, Oon Tek Ng

**Affiliations:** 1 National Centre for Infectious Diseases, Singapore, Singapore; 2 Department of Infectious Diseases, Tan Tock Seng Hospital, Singapore, Singapore; 3 DSO National Laboratories, Singapore, Singapore; 4 Lee Kong Chian School of Medicine, Nanyang Technological University, Singapore, Singapore; 5 Department of Laboratory Medicine, Tan Tock Seng Hospital, Singapore, Singapore; 6 Yong Loo Lin School of Medicine, National University of Singapore, Singapore, Singapore

Local transmission of severe acute respiratory syndrome coronavirus 2 (SARS-CoV-2) virus in Singapore has been reported.^1^ As the pandemic spreads globally, increased utilization and shortages of personal protective equipment (PPE) are expected. Although extended PPE use would mitigate utilization rate, its safety is unknown. At the National Centre for Infectious Diseases, recommendations for healthcare workers (HCWs) in contact with known or suspected patients are in concordance with the US Centers for Disease Control and Prevention, which recommends gloves, gown, respiratory protection (eg, disposable N95 respirator), and eye protection (eg, goggles or disposable face shield), without the use of shoe covers.^2^

An initial pilot study showed no contamination of N95 and disposable face visors after patient care, although in 1 instance, SARS-CoV-2 nucleic acid was detected on the front surface of an HCW’s shoe.^3^ To evaluate the safety of extended PPE use, we conducted a 1-day PPE sampling study on HCWs caring for confirmed COVID-19 patients to ascertain the per contact episode risk of PPE contamination with SARS-CoV-2.

## Methods

The PPE samples were collected by 5 trained personnel using a standardized technique with Puritan EnviroMax Plus premoistened sterile swabs (Puritan Medical Products, Guilford, ME) from the entire front of goggles, the front surface of N95 respirator, and the front surfaces of shoes of 30 HCWs (Table 1) exiting patient rooms. Gloves and gowns were not swabbed because these are disposed after each use. Data on HCW category and details of activity in the room were recorded. Patients with positive SARS-CoV-2 PCR assays within the prior 48 hours were selected, and clinical data (ie, day of illness, presence of symptoms, and cycle threshold [Ct] value of clinical PCR) were obtained from the medical record. Environmental samples were tested using specific real-time RT-PCR methods targeting the SARS-CoV-2 RNA-dependent RNA polymerase (RdRP) and E genes.^4^

**Table 1.** Characteristics of PPE Samples Collected and Relevant Patient Clinical Data

Sample No.	Staff Type	Duration of Time, Minutes	Activity	Clinical Data of Patient		
				Day of Illness	Symptomatic	Ct Value
1	Doctor	5	Examination	14	No	31.59
2	Doctor	5	Examination	9	Yes	20.80
3	Doctor	10	Communication without examination	9	Yes	20.80
4	Doctor	25	Examination	4	Yes	27.69
5	Doctor	6	Examination	8	Yes	30.7
6	Doctor	6	Examination	15	Yes	29.51
7	Doctor	8	Examination	19	Yes	30.24
8	Doctor	3	Examination	19	No	29.86
9	Doctor	11	Examination	15	No	31.4
10	Doctor	7	Examination	18	No	28.32
11	Doctor	5	Examination	14	Yes	29.02
12	Doctor	15	Examination	8	Yes	27.86
13	Doctor	20	Examination	8	Yes	27.86
14	Doctor	6	Examination	12	Yes	36.95
15	Doctor	10	Examination	10	No	31.33
16	Nurse	7	Collecting respiratory specimen	14	No	31.59
17	Nurse	5	Administering medications and communicating with patient	4	Yes	27.69
18	Nurse	18	Blood taking and communicating with patient	8	Yes	30.7
19	Nurse	19	Blood taking and collecting respiratory specimen	8	Yes	30.7
20	Nurse	4	Changing of wrist tag and collection of stool sample	15	Yes	29.51
21	Nurse	5	Collecting respiratory sample	18	No	28.32
22	Nurse	7	Collecting respiratory sample	19	Yes	30.24
23	Nurse	10	Administering medications	8	Yes	27.86
24	Nurse	5	Administering medications	20	Yes	29.91
25	Nurse	5	Monitoring vitals	15	Yes	32.23
26	Cleaner	5	Cleaning of high-touch areas	14	No	31.59
27	Cleaner	7	Cleaning of high-touch areas	9	Yes	20.80
28	Cleaner	2	Clearing trash	18	No	28.32
29	Cleaner	3	Clearing trash	15	No	31.4
30	Cleaner	3	Clearing trash	19	No	29.86

Note. Ct, cycle threshold. Cycle threshold refers to the number of cycles required for the fluorescent signal to cross the threshold in RT-PCR; a lower cycle threshold value indicates a higher viral load.

## Results

In total, 15 patients (7 women and 8 men) were selected. Patient characteristics varied by day of illness (median, day 14; interquartile range [IQR], 8.25–17.25), presence of symptoms (63% symptomatic), and clinical PCR Ct value (median, 30.08; IQR 28.85–30.86). No patient required ventilatory support and no aerosol-generating procedures were carried out prior to or during sampling. All 90 samples from 30 HCWs (doctors, nurses, and cleaners) were negative (Table 1). The median time spent in the patient’s room overall was 6 minutes (IQR, 5–10): 8 minutes for doctors, 7 minutes for nurses, and 3 minutes for cleaning staff. Activities ranged from casual contact (eg, administering medications or cleaning) to closer contact (eg, physical examination or collection of respiratory samples).

## Discussion

Our study had several limitations. One limitation of our study was the use of surface swabs for sampling the surface of N95 masks rather than processing masks in extraction buffers with detergents, which is a method that has been used for isolation of influenza from N95 respirators.^5^ Surface swabbing may be insufficient for the detection of entrapped viral particles. Second, all patients were in airborne infection isolation rooms with 12 air exchanges per hour, and these results may not be generalizable to other room configurations. Third, we did not assess the concomitant level of viral contamination of the environment in this study to correlate with the level of PPE contamination.

Previous laboratory studies have demonstrated that viruses, such as SARS-CoV and human coronavirus 229E, can remain viable on PPE items, including latex gloves and disposable gowns,^6–8^ but these studies were not performed in clinical settings. Despite the potential for extensive environmental contamination by SARS-CoV-2, we did not find similar contamination of PPE after patient contact. These results provide assurance that extended use of N95 and goggles with strict adherence to environmental and hand hygiene while managing SARS-CoV-2 patients could be a safe option.
